# Temporal Dynamics With and Without a Nervous System: Plant Physiology, Communication, and Movement

**DOI:** 10.1111/cogs.70079

**Published:** 2025-06-23

**Authors:** Margherita Bianchi, Silvia Guerra, Bianca Bonato, Sara Avesani, Laura Ravazzolo, Valentina Simonetti, Marco Dadda, Umberto Castiello

**Affiliations:** ^1^ Department of General Psychology University of Padua; ^2^ DAFNAE‐Department of Agronomy, Food, Natural Resources, Animals and Environment University of Padua

**Keywords:** Time perception, Philosophy of time, Temporal processes, Plant physiology and behavior, Plant signaling, Plant movement, Comparative cognition, Ecological communication, Kinematics, Isochrony

## Abstract

The concept of time has long been the subject of complex philosophical reflections and scientific research, which have interpreted it differently based on the starting question, context, and level of analysis of the system under investigation. In the present review, we first explore how time has been studied among different scientific fields such as physics, neuroscience, and bioecological sciences. We emphasize the fundamental role of an organism's ability to perceive the passage of time and dynamically adapt to its environment for survival. Growth, reproduction, and communication processes are subject to spatiotemporal variability, and the sense of time allows organisms to structure their interactions, track past, and anticipate future events. Specifically, building on a relational and multilevel approach, this paper proposes an analysis of various aspects of the temporal dimension of plants—ranging from their growth and adaptation rates to behavioral strategies and modes of communication—culminating in a focused examination of research based on the kinematical analysis of plant movement. By adopting a comparative and critical approach, we raise several questions about the temporality of processes from different perspectives. Further insights into the timing of physiological and communication processes in plants will help to recognize the central role of temporality in life and to discover mechanisms, processes, and behavioral strategies that may be common (or similar) across species or unique (species‐specific) for some organisms, both with and without nervous systems.

## 1 The concept of time: Bridging philosophy and science

Since ancient times, the concept of time has been the central focus of numerous philosophical reflections and scientific investigations (cf. Arstila & Lloyd, 2021; Bardon, 2024), which have, in different ways, highlighted its complexity (cf. Griffin, 1985). Indeed, time has been considered both an aspect of physical reality, an objective substance, a relational emergence, and an experience of psychological re‐elaboration.

As Guido Tonelli (2023, p. 29) wrote in his book dedicated to a detailed analysis of the concept of time, it is already in ancient Greece that several terms and related concepts were used to describe “time.” For example, *Témno* referred to the act of cutting or dividing, suggesting the separation of an interval; *Chrónos* referred to chronological or sequential time; *Aión* denoted eternal or metaphysical time; and *Kairós* indicated the opportune or right moment to act.

Nowadays, as Souza, Posso, and de Carvalho Oliveira (2024) have pointed out, time is conceived in different ways, such as (1) a general phenomenon that governs the succession of events, in which there are cause relationships that precede the effects; (2) an interval within a larger succession of events; (3) the duration of a specific event; and (4) a specific moment of the beginning or occurrence of an event.

Before becoming the subject of scientific investigation, the idea of time, often linked to that of space and movement, was explored by numerous philosophers. For example, Aristotle did not consider time an independent substance, but rather a measure of change. Lucretius envisioned time as intrinsically linked to motion, which unfolds within space. For Saint Augustine, the concept of time was closely tied to the human mind's capacity to perceive temporal intervals, famously asserting that we know what time is, until we try to explain it. For Leibniz, time represented the order of succession (just as space represented the order of coexistence). Kant regarded time, alongside space, as an a priori form of human intuition, structuring how we perceive phenomena. Bergson emphasized a dynamic, qualitative, and subjective experience of time. The collection of these positions has formed the foundation for subsequent research on time, conducted at various levels of analysis and across different fields (e.g., in physics, psychology, and literature).

Currently, there is no single meaning and use of the term “time” (cf. Rovelli, 2017; 2020), and several questions arise regarding what time is and what our relationship with it is. Examples are those relating to our efforts to control time, to reduce its progression, and the awareness that it is impossible to stop it. Other reflections concern the human desire to reverse the course of time: to invert its directional arrow (cf. Wallace, 2013). The idea of “reversing time” takes on different meanings depending on whether we refer to a mental operation—through which we do not alter the past itself, but reinterpret our idea of it in the present—or to events in the physical world, where it is not conceivable that an action performed today could modify what has already occurred. Every system, even nonliving ones, undergoes changes and does not remain entirely identical to itself over time and space. Even in a simple system, consisting of a few elements, one could get closer to the initial configuration, but it would not be a real turning back in time, since it would be a new action and one would still move forward. In macroscopic reality, irreversibility emerges: the arrow of time becomes evident through the increase of entropy and the impossibility of perfectly restoring a previous state (cf. Tonelli, 2023, pp. 116; 169).

Other questions are ontological‐epistemological, relating to the fact that time has its own and separate existence or is rather a great “illusion,” or it is better conceived as a useful tool for understanding some aspects of reality, especially for controlling the organization of human societies (cf. Orzel, 2022). From this perspective, the functional subdivision between past, present, and future constituted the pillars thanks to which it was possible to plan and organize the first civilizations, and which are still the basis of human living practices. As evidenced by the widespread presence of diverse types of clocks, time marks the rhythm of the days and the division of our commitments (Tonelli, 2023, pp. 16; 28; 44).

### 1.1 The diversity of time: Micro and macro characteristics

A key point that needs clarification is that time has different characteristics depending on whether it is considered at a microscopic (i.e., the interaction of individual particles) or macroscopic level (i.e., the collective properties of large systems emerging from microscopic interactions) (cf. Rovelli, 2018).

The widespread habit of conceiving the passage of time as a regular phenomenon derives from the development of our possibilities of life in an area of the Universe characterized by periodic and regular, rather than turbulent and chaotic processes. Indeed, diverse types of entities and processes are subject to the passage of time. Time acquires different characteristics depending on the scale of analysis adopted. Think, for example, of the analysis of phenomena that occur at the subnuclear level, such as the “life or death processes” of subatomic particles—which have a constant probability of decaying over time (immediate disintegration or survival of an unimaginable duration if compared to that of the longest‐lived living beings (cf. Pineau et al., 2024)—or, conversely, to those concerning gigantic entities such as galaxy clusters (Tonelli, 2023, p. 16; 24–25; 126).

Even remaining in the field of physics, it is possible to observe the transformation of theories that have thematized and used the concept of time differently (cf. DiSalle, 2006). For example, in the Newtonian conception of the laws of motion, space and time are conceived as absolute axioms, that is, as an immutable background against which movements stand out. This conception of absolute time has been questioned over the last century, particularly since Einstein showed that continuing to consider time as “absolutus,” which proceeds at a fixed speed independent of matter, would have led to paradoxes. Time is not universal and identical for all observers, but relative to the frame of reference. Special relativity introduced the concept of space‐time, a continuous, four‐dimensional structure in which space and time are inseparable. In general relativity, time can also be deformed: masses and energies curve space‐time, locally influencing the flow of time, which slows down near stronger gravitational fields. Moreover, it is not necessarily the case that time has always “existed.” In 1927, Lemaitre hypothesized that the universe expands, and galaxies move away at a speed proportional to their distance: this is the origin of what we now call the “Big Bang” theory (cf. Uzan, 2021). Lemaitre's considerations have been confirmed by Hubble's telescopic observations, although it is not the galaxies that move, but space‐time that expands. Today, we know that space‐time had a beginning (about 13.8 billion years ago), and its accelerated expansion is attributed to dark energy (Tonelli, 2023, pp. 45–54; 65–70).

Currently, there are various proposals to reconcile relativity and quantum mechanics. Within contemporary approaches to quantum gravity, some authors have questioned the necessity of time as a fundamental variable, suggesting it might be an emergent or even illusory phenomenon. This line of thought has also been extended to space, particularly in approaches where spatial geometry is hypothesized to emerge from patterns of quantum entanglement (cf. Van Raamsdonk, 2010). For example, in the Wheeler–DeWitt equation, time does not explicitly appear. In Loop Quantum Gravity, the focus shifts to the quantum properties of space‐time itself, which is conceived as a finely granular structure. On infinitesimal scales, space would not be continuous, but composed of a discrete network of quantized units, often represented mathematically as spin networks (cf. Rovelli, 2018). These ideas remain theoretical and await experimental confirmation (Tonelli, 2023, pp. 175–180).

### 1.2 A web of relationships: Events that connect to other events

Beyond the future of the above‐mentioned theories, which can be verified or falsified, the interesting aspect is that the universe is the result of events that relate to other events, constituting a complex network of relationships. At stake is a relational conception: time and space would arise from the interaction of elements (cf. Bianchi, 2022).

Time, therefore, would not be denied at any level of analysis (e.g., at the psychological level or at the level of the organization of living systems); rather, it would be a matter of adopting a different way of reasoning, one based on relational terms. Events would arise from the relationships they establish (cf. Whitehead, 2010).

Therefore, in this analysis, it is essential to identify the main question to answer and the appropriate level of analysis for the aspect of reality being investigated, considering one's cognitive limits and methodological‐observational capabilities (cf. Bianchi et al., 2025).

For instance, adopting a relational, systemic, and process‐oriented approach to studying biological phenomena—now increasingly encompassing behavioral and cognitive dimensions—may enhance our ability to analyze natural phenomena.

For the purposes of this paper, this perspective could help to integrate and more accurately understand plant‐specific characteristics within the broader framework of studies on behavior and cognition (cf. Bianchi, 2022). In this context, particular attention is given to the temporality of processes, useful in explaining the transformation of behavioral and communication strategies during the individual development of organisms, in their living contexts, in which other organisms, with similar or very different ecological needs and life spans, are also present. An integrated analysis of the relevance of time in the study of life will help to provide a more unified and informative picture of the changing relationships and behavior across *taxa* (cf. Bianchi, 2024b).

Therefore, before delving into the question of time in relation to the plant world, it is necessary to clarify what time means to us, at both a subjective and neurological level, and, from a broader perspective, its role at a bioecological level, in the organization of living systems (cf. Ng, Garcia, Dyer, & Stuart‐Fox, 2020).

## 2 Biological time in comparative studies on behavior and cognition

In this section, we focus on studies conducted on humans and other animals. We address this issue at first from a neuroscientific and psychological point of view, and then from a bioecological perspective. We will emphasize the importance of time in the life of organisms for communication and survival in general, highlighting its variability in the various *taxa*, characterized by different body organizations and both similar and different ecological needs.

### 2.1 The perception of time in cognitive science

We begin by examining the concept of time in human cognition, a subject explored by many thinkers throughout history. Among the most renowned was Augustine, who conceived time as a succession of states of consciousness that we perceive and measure. The present, the past, and the future exist only in our “soul”: their reality depends on human perception. According to the author, if we think at a specific moment in the past, that moment no longer exists; the future has to come and the present is translated moment by moment into the past (otherwise, it would be “eternity”) (cf. Saint Augustine, 2017).

Concerning the perception of time, as investigated in the context of contemporary neuroscience, considerable progress has been made in understanding the processes underlying the ability to “feel” time and in general its indispensability for people's lives (cf. Buonomano, 2017; Wittmann, 2016).

We now know that the processes of human life, both in the awake state and during sleep, are based on some temporal organization (cf. Wright, Lowry, & LeBourgeois, 2012). Indeed, memories are embedded in the space and time in which they were constructed. The brain elaborates temporal processes even in the absence of external stimuli, and even our dreams sometimes follow a temporal order (cf. Northoff, Scalabrini, & Fogel, 2023).

As far as subjective time, linked to individual perception, is concerned, an extremely fascinating aspect, that gives an idea of functional processing a posteriori, is the illusion of sensory simultaneity at the level of conscious perception. Given the different speeds between diverse sensory signals (e.g., visual and tactile), we do not live in a real “present” but in a past present of about half a second, reworked by the brain (cf. Hogendoorn, 2022). The brain integrates and compensates for temporal and spatial discrepancies in sensory input, constructing a coherent and unified perception of the world, essential for guiding action and behavior. Therefore, what we consider the “present” is, in a certain sense, an artificial construct, just like the past, shaped and partly modified by the plastic action of memory (cf. Petrucci & Palombo, 2021). Dialogue with the future also has a shaping action on the present. Therefore, it seems that emotions and memories play a decisive role in the construction of the sense of time in human beings, endowed with a rich inner life.

Moreover, on a subjective level, several factors and experiences can alter and deform the sense of time: if we are relaxed or having fun, it seems to flow faster, while in situations of stress or danger, it seems to slow down (cf. Pfeifer, Pothmann, Claaßen, & Wittmann, 2023). Individual and situational differences in temporal perception correlate perception and action (cf. Keele, Pokorny, Corcos, & Ivry, 1985; Morin & Grondin; 2024; Moore & Olson, 2024). The temporal “representation,” therefore, also depends on the context (cf. Karmarkar & Buonomano, 2007) and can be conditioned by various factors external to the organization of the system in question.

In addition, at the level of temporal perception, time distortion phenomena have been observed in humans, influenced by different types of expectations. For example, Ivry and Schlerf (2008) have demonstrated that when a stream of digits is presented with the same time duration, and the same digit is presented repeatedly, the initial movement is perceived as having a longer duration. Similarly, if a set of digits is presented in their standard ordinal position (e.g., 1 2 3 4): the “1” is perceived as longer than the “2,” “3,” and “4,” while if the order is different and kept hidden (e.g., 1 4 3 2), there is no distortion of duration. This signifies that events capturing the most attention are those perceived as having a longer duration (cf. Tse, Intriligator, Rivest, & Cavanagh, 2004).

Regarding the sensory capacities involved in the perception of time, the “sense” of time involves numerous processes: it is not linked to the presence of a single specialized organ (Ivry & Schlerf, 2008). Therefore, excluding an atomistic or isolated analysis, problems of “localization” are avoided, referring to the joint activity of a network of processes (cf. Lewis & Miall, 2003).

Even in the human brain itself, different regions are involved in temporal perception, in the evaluation of the expectation of events, their sequencing, and spatial ordering (Tonelli, 2023, pp. 31–32). In humans, the brain appears to play the most crucial role in temporal awareness. This has been revealed in a series of studies conducted on individuals who have suffered serious brain injuries (e.g., accidents or brain tumors) and who have consequently manifested significant alterations or losses in the sense of time, which has made it difficult or impossible to carry on their lives independently (cf. Gazzaniga, 2012). It should be noted, however, that not only the brain is involved in temporal perception but also the rest of the body, in relation to the environment. It is, therefore, a complex set of processes in which the whole body is used (Rusinova, Volodina, Terenteva, & Kosonogov, 2024). Some senses, depending on the species considered and the tasks performed, could have a predominance over others in the encoding and perception of temporal information (e.g., the sense of hearing) (cf. Guttman, Gilroy, & Blake, 2005).

These considerations raise interest in the processes underlying temporal perception in organisms with different body structures, organizations, and ecological needs.

### 2.2 Time in the biological sciences

We can now ask ourselves what makes the ability to perceive time and the possibility, even subconscious, of ordering behaviors in diachronic sequences. Why, then, is time so relevant for survival?

First, it is important to recall a fundamental characteristic of biological systems: they are thermodynamically open systems, exchanging energy and matter with their environment. While local decreases in entropy can occur, as seen in the complex organization of living matter, these are always accompanied by a greater increase in the entropy of the surroundings. In this way, the microscopic physical dynamics give rise to the macroscopic processes that govern the evolution of material bodies, including living organisms (cf. Tonelli, 2023, pp. 161–164; 178). This aspect creates useful connections to describe the development of behavioral and cognitive dynamics of living systems, even in the simplest ones, over time and space, thanks to the ability to keep track of what has happened and to predict what will happen through anticipatory behaviors (cf. Deans, 2021). The directionality of time affects the behavioral and cognitive levels of organisms, and it can be conceived as a chronological process, allowing them to solve survival problems (cf. Novoplansky, 2015; Bianchi, 2024b). In systemic approaches and complexity science (cf. Wegner, 2024), time is often conceived as an emergent property, arising functionally from the nonlinear interactions among a system's components, as it operates under constant flows of energy and matter exchanged with its environment (Souza et al., 2024). Even the metabolic organization of organisms—metabolism in a broad sense, referring to the totality of biochemical transformations underlying the living activities of organisms—underlies these temporally cyclical but ultimately continuous dynamics. For organisms’ metabolic cycles to persist, it is necessary to renew and keep active the series of processes that make up systems (cf. Keijzer, 2020). The organization of this series of processes takes shape, is maintained, and evolves over time within the environment (cf. Maturana & Varela, 2012). Living organisms can be understood as open thermodynamic systems operating far from static equilibrium (Souza & Lüttge, 2014).

From a biological and ecological point of view, we will now see why various species need time to survive. Starting from the most basic, often automatic and unconscious bodily processes that make life possible, it is important to note that there is generally a periodic (or cyclical) pattern, which is temporally marked. Examples are heartbeat, breathing, and the alternation of sleep and wakefulness. The importance of the regularity of these processes becomes evident when disruptions occur, impairing their function. One of the most extensively studied processes in biological systems—since its first measurement in 1729 by De Mairan (cf. McClung, Salomé, & Michael, 2002)—is the circadian cycle (cf. Gardner, Hubbard, Hotta, Dodd, & Webb, 2006). At the core of these processes, there are biological clocks, that is, internal biochemical mechanisms of cells, which act to adapt living systems to the changes produced by the Earth's rotation. In different species, many activities are synchronized with the day/night light cycle. Overall, the metabolism and behavior of living beings are synchronized over a cycle of about 24 h and most of the physiological processes are programmed to be more active during the day, while others are carried out more efficiently in the absence of light. Regarding the cellular basis of time perception, as proposed by Baluška and Reber (2021), living organisms, both unicellular and multicellular, possess circadian biological clocks. These are ancient anticipatory systems that coevolved with the earliest cells to preserve their integrity, enhancing the cell's ability to functionally respond to its environment by anticipating external regularities such as gravity, light, and temperature. This anticipatory capacity enables appropriate behavioral responses and, at the supracellular level, supports the survival of the organism. Circadian clocks represent a bottom‐up approach to time perception in living systems. The integration of cellular circadian clocks and cellular sentience is essential to the cognitive basis of life.

From a strict behavioral perspective, the sense of time serves organisms to adapt their behaviors to respond adequately to transformations that take place in their environments, as light changes. This is a fundamental skill for navigating the world and generally for preparing to act. Organisms need it to causally link events together to build an action sequence for exploiting opportunities and avoiding hazards and threats in their environment. A living being unable to keep track of the passage of time could not regulate its behavior, considering that all events occur at a given moment, for a finite duration, and in a certain sequential order, perceived and identified by a specific organism based on its ecological needs (cf. Parent, Takasu, Brodeur, & Boivin, 2017).

A concrete example related to the ability to perceive the passage of time is linked to foraging dynamics (cf. Charnov, 1976); specifically, to the possibility of organisms procuring, storing, and recovering food brought and stored in different places (cf. Clayton & Dickinson, 1999).

Although the ability of organisms to measure time and consequently regulate their behavior has been studied, in natural and experimental contexts, mainly in humans and other animal species, especially in vertebrates (cf. Babb & Crystal 2005; Roberts & Feeney, 2009), more recently, this investigation has been extended to invertebrates such as cephalopods (Jozet‐Alves, Bertin, & Clayton, 2013) and various species of insects (Clayton & Dickinson, 1998; De & Chatterjee, 2021).

The set of these comparative studies on the sense of time (and space) will greatly help to better understand some of the elementary mechanisms underlying the strategies, which permit organizing and optimizing resources and energy expenditure, and to apply more effectively various forms of learning and communication.

To integrate and expand this analysis, it is essential to include and explore these aspects in relation to the plant kingdom.

## 3 The need to consider time in plant sciences

As highlighted by Souza et al. (2024), although the fact that the plant dimension is implicitly and fundamentally focused on understanding the evolutionary and adaptive processes of plants over time, plant physiology has been based on methods which have tended to neglect the temporal element.

In addition to the complexity of the phenomena, and beyond the practical constraints, the concept of time, in its various theoretical and applied facets, plays a fundamental role in achieving a more complete and realistic understanding of plant processes dynamics and the range of behaviors that are being increasingly studied (cf. Calvo & Trewavas, 2021).

Therefore, an epistemological analysis should allow us to examine plant biology as a set of processes that take place over time, paying adequate attention to the interconnection of events inside and outside plant bodies. In this perspective, processual approaches provide an adequate basis for the development of a plant science focused on the interconnected analysis of its processes (cf. Bianchi, 2022), paying more attention to the behavioral dimension, both individual (pertaining to a single plant) and social (involving multiple interacting plants), both intra and interspecific (within and between species).

The following section is specifically dedicated to the issue of time in the plant kingdom. We shall focus on plant physiology and, subsequently, on the aspect of temporality emerging from studies on plant communication and behavior.

### 3.1 Time in plants physiology: Growth, adaptation, and reproduction

The need to explore temporal aspects in adaptive eco‐evolutionary dynamics and plant physiology is increasingly recognized (Souza et al., 2024). This stems from the idea of a multielements processual approach, leading to specific outcomes occurring in a given time and context, remaining capable of further development (cf. Nicholson & Dupré, 2018).

From this perspective, living systems—both neural and aneural—can be seen as spatiotemporal dynamic entities, whose possibilities of interaction and communication are realized within certain constraints. Their continuity over time is essential for understanding them as persistent metabolic and functional‐agentive entities. Organisms are not static but rather complex networks of interconnected processes, based on signaling pathways, which are constantly transforming (cf. Bianchi & Castiello, 2023a; 2023b) in which something is preserved and repeated (cf. De la Fuente, 2015) and something is changed more flexibly (cf. De Kroon, Visser, Huber, Mommer, & Hutchings, 2009). Every organism, regardless of the timescale on which it operates, possesses the capacity to generate effective responses by structuring its interactions, retaining acquired information, and anticipating future events. Although the results of the so‐called “omics” sciences (metabolomics, proteomics, transcriptomics, phenomics, etc.) are significant and increasing (cf. Alberghina, Hohmann, & Westerhoff, 2005; Amaral & Souza, 2017; Tolani, Gupta, Yadav, Aggarwal, & Yadav, 2021), much remains to be explored about the different times of plants life, during their individual and intergenerational development.

**Fig. 1:**
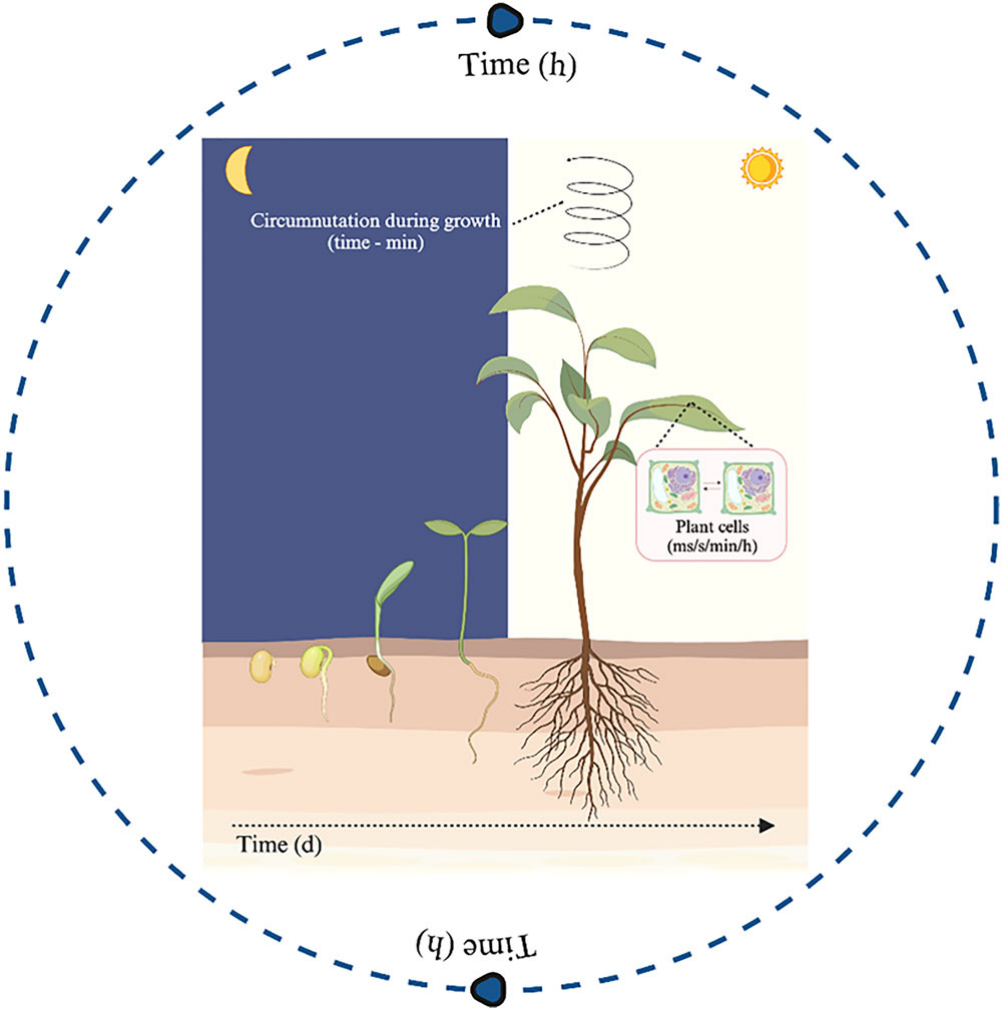
The timing of plant physiological processes. Graphical representation of the physiological mechanisms and processes that enable plants to anticipate environmental changes and regulate their vital cycles, from the intra‐ and intercellular communication of plant cells to the growth of the plant. Plant biological rhythms can be detected and measured over a wide range of timescales, from milliseconds to days. Abbreviations: d, days; h, hours; min, minutes; ms, milliseconds; s, seconds.

Starting from the most basic processes, plants, like all other eukaryotes and most prokaryotes, are able to “read the time,” even in the absence of a nervous system. They are equipped with molecular mechanisms that allow them to anticipate predictable changes in the environment. Many aspects of metabolic regulation and plant development are governed by circadian rhythms, that is, interconnected feedback loops (Gardner et al., 2006). Even in plants, these mechanisms have evolved because temporal programming offers selective and bioecological advantages (Srivastava et al., 2019). A functional circadian clock, synchronized with the day‐night cycle, enhances carbon fixation through photosynthesis, facilitating plant growth (cf. Greenwood & Locke, 2020; Fig. 1).

As highlighted by Baluška and Reber (2021), temporal regulation in plant systems reflects a decentralized organization, in which different organs can exhibit autonomous rhythmic activity based on the integration of local cellular clocks. Plant circadian clocks are organized in a tissue‐specific and interorgan manner, with cell‐to‐cell coupling likely mediated by chemical and electrical signals (cf. McClung, 2006). Isolated organs, such as leaves and roots, are capable of maintaining their own circadian rhythms as long as they remain viable.

By anticipating environmental changes, plants reduce the delay between the occurrence of a change and the corresponding physiological response (cf. Dodd et al., 2005). These mechanisms control various plant biological processes such as photosynthesis, respiration, seed germination, hypocotyl elongation, leaf movement, stomatal conductance, and senescence processes. Thus, overall, circadian clocks, responsible for regulating cyclical changes in the environment, underlie daily and seasonal variations, and the rhythmicity of the biological organization of plants, which includes a wide spectrum of times ranging from fractions of seconds, such as photochemical events, to annual cycles, as exemplified by the annual flowering of numerous tree species (Souza et al., 2024; Fig. 1).

It is, therefore, understandable how times and rhythms characterizing plant life vary according to the elements considered. For instance, regarding time as duration, the seed germination process can vary significantly, from days to months, depending on several factors, such as a species’ ability to absorb water, to perceive and process signals, to suppress seed dormancy in specific cases (Fig. 1). The “opportune time” in plant species reproductive success depends on flowering at the right time (Putterill, Laurie, & Macknight, 2004).

**Fig. 2:**
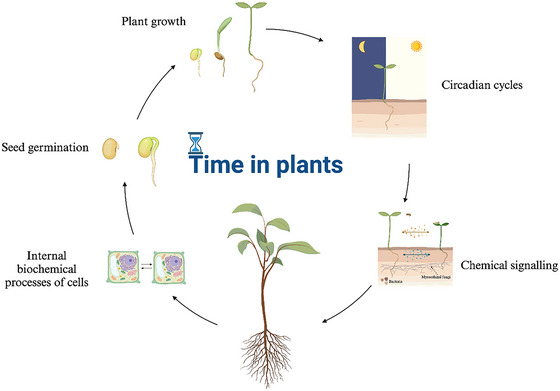
Multilevel analysis of time in plant life. Graphical representation of the temporal dimension of plants at different growth stages (e.g., seed germination) and during internal (e.g., circadian cycle) and external processes (e.g., communication with other organisms).

Similarly, the concept of growth is not unambiguous, as its meaning varies depending on the timescale and context. As Hilty, Muller, Pantin, and Leuzinger (2021) highlighted, in plant science and ecology, “growth” is a widely used term that can take on different interpretations depending on the spatiotemporal scale and organizational context of the analyzed system. The challenges inherent in growth studies include the observation and quantification of processes that range over different orders of magnitude from the cell to the community, ranging from minutes to centuries, as in the case of immediate stress responses or ecological successions, respectively (Fig. 2).

At the cellular level, growth is often associated with meristematic activity that produces cells and initiates new organs (Fig. 2). At the organ level, growth is concerned with the elongation or expansion of roots, stems, and leaves. At the plant level, over longer periods, the increase in biomass is measured. At the ecosystem level, growth is often conceptualized as the accumulation of biomass per unit of land area and time. When the focus shifts from individual plants to communities, the Earth's surface becomes an additional reference metric. The various aspects of growth, therefore, range from cells to organs, plants, and ecosystems: all this represents the “what.” Instead, the measurement methods suitable for analysis concern the “how,” and finally, the reason to distinguish different aspects of growth, the “why” (Hilty et al., 2021). For example, distinguishing between different aspects of growth and studying their coupling is crucial to identify what causes, contributes to (or limits) plant growth, an aspect that has implications for understanding ecosystem well‐being and human health itself (cf. Bianchi, 2021). The analysis of these different spatiotemporal scales refers to partially overlapping processes, not clearly distinct, but distinguishable by us (Fig. 2).

Overall, the combination of these processes forms the basis of varied lifespan and periods of plant reproduction. In the animal kingdom, Charnov's (1991) model distinguished between organisms that live at a fast rate with high mortality and short age up to sexual maturity and those that lead “slower” ways of life, with low mortality and long age up to sexual maturity. Franco and Silvertown (1996) explored the hypothesis of the fast‐slow continuum in plants. In line with the results observed in the animal world, a fast‐slow continuum was found: even plants that suffer high adult mortality have a shorter lifespan and reach sexual maturity at an earlier age. However, unlike many animals, plants endowed with meristems and high phenotypic plasticity can increase their size and, consequently, their fecundity, partly counterbalancing the effect of mortality. These differences mainly arise from the different modular structures of plants, where functions are distributed.

From a comparative perspective, these (cautious) comparisons between animal and plant characteristics will help deepen our understanding of how different degrees of morphological and physiological integration affect the times of reproduction, life, and ecological interactions of plants. This, in turn, facilitates a better understanding of how unique these organisms are and how much they share with the animal kingdom.

A point worth noting is that plants cope with environmental variability perceiving the duration and the regularity of various external signals to adapt their responses (Trewavas, 2014). A fundamental aspect of plant life is their ability to perceive different wavelengths of the electromagnetic spectrum to obtain energy and synthesize compounds essential for building their bodies. Plants are indeed equipped with a series of photoreceptors and related signaling pathways to transmit the light information derived from the environment (Sanchez, Rugnone, & Kay, 2020).

### 3.2 Temporal dynamics of plant signaling and communication

At a fundamental level of signaling, plants are capable of both detecting and emitting chemical compounds—through the air via volatile organic compounds (VOCs) and in the soil through root exudates—thereby enabling interactions with other plants, mycorrhizal fungi, and microorganisms such as bacteria (Fig. 2). Many forms of signaling occur within plant bodies, others externally via VOCs over short distances, between adjacent plants (cf. Karban, 2021; Yoneya & Takabayashi, 2014).

It is worth noting that plant life, which may appear uniformly slow to the human eye, is in fact based on signaling processes of variable duration, which can be empirically observed and measured. Signaling times vary significantly depending on the type of stimulus, the distance involved, and the transmission medium (cf. Trewavas, 2007). For example, electrical signaling in plants occurs within fractions of a second to a few seconds (cf. Szechyńska‐Hebda et al., 2022; Zimmermann, & Mithöfer, 2013). Signals such as changes in pressure or cellular turgor in response to environmental stimuli typically take several seconds to a few minutes (cf. Fricke, 2017). Hormonal signaling generally unfolds over the course of minutes to hours (cf. Moore et al., 2022; Weyers & Paterson, 2001). Thus, plants are capable of signaling and communicative responses that may vary in speed but are effective in addressing their diverse ecological demands.

More specifically, regarding chemical signaling, it can mediate both direct and indirect defense responses to herbivore and pathogen attacks, with varying intensity depending on the type, concentration, and duration of exposure to a given substance (Yi, Heil, Adame‐Álvarez, Ballhorn, & Ryu, 2009). To better understand how long and at what concentration a plant should be exposed to a volatile compound to elicit a detectable response, Girón‐Calva, Molina‐Torres, and Heil (2012) examined the effect of different concentrations of nonanal and methyl salicylate (MeSA) on Lima bean (*Phaseolus lunatus* L.) for 6 or 24 h. Both compounds enhance the plant's resistance to the bacterial pathogen *Pseudomonas syringae* pv. syringae, at both the phenotypic and gene expression levels. Exposure to nonanal for 6 h significantly increased resistance, an effect that persisted even after 24 h. In contrast, a low concentration of MeSA had no significant effect after 6 h, while after 24 h, it significantly improved resistance to the pathogen. This indicates that there is a relationship between dose, exposure, and plant response (Girón‐Calva et al., 2012).

Thus, the temporal dimension represents an important element in the conception and differentiation of biological stress, which takes into account past experiences to initiate and calibrate defense and repair responses to actual or potential damage (Galviz, Souza, & Lüttge, 2022). This leads us to reflect on the fact that the stress a plant experiences during its development is not a single moment in its life; instead, it is a dynamic process influenced by both the plant's current condition and past events. For this reason, each individual life path is unique and constantly subject to change (cf. Bianchi, 2022).

Moreover, reflecting on signaling and communication processes within ecosystems allows for a deeper understanding of how essential relationships among organisms—from different species and kingdoms—are shaped and structured across both shared and distinct temporal scales (cf. Payrató‐Borràs, Gracia‐Lázaro, Hernández, & Moreno, 2024; Tylianakis & Morris, 2017). These processes do not always occur or unfold simultaneously. Physical and bioecological timescales intersect within a common temporal framework, yet follow species‐specific rhythms and dynamics. Understanding these varied temporalities is fundamental for deepening our awareness of what may be called a shared “ecological time” (cf. Bianchi, 2024b).

### 3.3 Time and movement in plant behavior

**Fig. 3:**
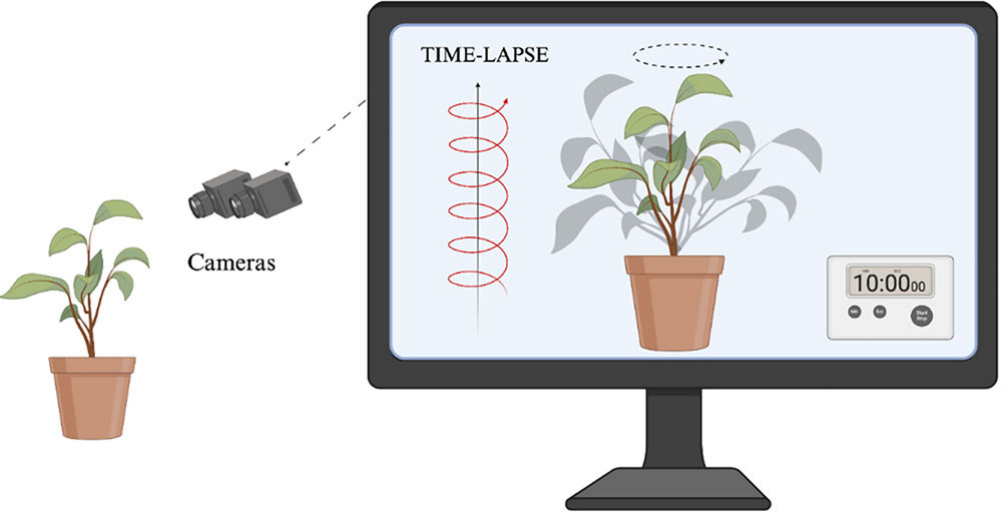
Plants’ time and our observational constraints. Graphical representation of the time‐lapse photography technique. Plants move on a different timescale to animals, including humans, making their movements invisible to the human eye. Using time‐lapse techniques, it is possible to speed up the recording of footage of plants to observe and study their movement and behavior.

Because plants move at a different timescale from the one we can perceive, they have represented a significant challenge to our possibilities of understanding their behavior (cf. Mancuso, 2017). Except for a few cases (e.g., plants capable of rapid movements such as *Dionaea muscipula* or *Mimosa pudica*), the movement of the plants is very slow and invisible to our eyes. Thanks to the introduction and dissemination of time‐lapse techniques (i.e., video assembled from consecutive images; Fig. 3), it has been possible to observe and study their different types of movements (cf. Castiello, 2019; Forterre, 2013), and consequently, a better understanding of their behavior (cf. Trewavas, 2014).

This aspect allows us to delve into the ecological significance of time for plant activities more deeply. The consideration of the temporal dimension can greatly contribute to understanding how plants interact, communicate, and solve survival problems in their living environment (Fig. 3).

In particular, the case of interest here is related to the perception and anticipation processes put in place by climbing plants to plan the ascent and attachment behavior to a potential support. More specifically, we refer to *Pisumsativum* L., which represents an excellent model because it is able to show rapid changes in response to environmental signals (cf. Gianoli, 2015). For these plants, finding adequate support is essential to develop in a sufficiently robust manner to access light and to improve their fitness (cf. Gianoli & Gonzalez‐Teuber, 2005).

Overall, this research has shed light on how sessile organisms without a nervous system are able to program and control their movement in response to different contexts.

As studied in our and other animal species, the execution of a reach toward and grasp of an object requires a series of time‐related processes that are strongly interrelated (cf. Castiello, 2005; Castiello & Dadda, 2019; Flash, Meirovitch, & Barliya, 2013). For instance, the time at which the maximum hand aperture is reached correlates with time events characterizing the deceleration phase of the arm accompanying the hand toward the object smoothly (Jeannerod, 1984). This relationship varies depending on object properties and context. In plants, these aspects have been studied by looking at the kinematics of pea plants’ tendrils during circumnutation, the ubiquitous pattern of oscillating growth displaced by elongating plant organs. That climbers are able to perceive and consequently modulate movement, based on the properties of a potential support, had already been noted by the pioneering studies of Francis and Charles Darwin (1875; 1880). Nowadays, these studies have been continued with advanced technological instrumentation, which allows more accurate observations, confirming that plants can achieve their survival goals in a targeted manner. Plants would seem to be able to plan and execute movements anticipating their effects in terms of time (Bonato et al., 2024).

Guerra et al. (2019) showed that pea plants can perceive and modulate the kinematics of the opening of tendrils depending on the thickness of the pole. Specifically, it has been observed that in the presence of a diameter beyond a certain size, climbers are unable to maintain the tension forces to adhere to the support, which is why they avoid twisting around it. It is a metabolic compromise adopted by plants, detected in their movement, which was slower toward thick rather than thin supports. Moreover, for the more difficult task (i.e., thicker supports), multiple online adjustments were measured. They were observable in the form of submovements along the velocity profiles, in line with the speed‐accuracy trade‐off principle (Fitts, 1954). This principle indicates that the time to perform an action is proportional to the information needed to regulate the movement. Plants were able to modify the speed and time of their reaching and grasping movements toward a support, modulating accuracy thanks to a series of submovements (Bonato et al., 2024; Ceccarini et al., 2020; 2021). Furthermore, it has been observed that this strategy is adopted by plants during their entire growth. Pea plants, indeed, tend to develop several leaves along the stem before the clasping of the support. Each leaf shows a helical movement allowing the plant to explore its surroundings to search a potential support upon which grows toward the light source. Recently, it has been demonstrated that as the number of leaves developed increases, the speed and time required to complete a movement increases. This corresponds to a decrease in the number of circumnutations and directional changes, which reaches its peak during the last leaf clasping the support. The progression and changes in the characteristics of exploratory movements of the leaves may allow plants to (1) accumulate the necessary energy and resources to implement the final launch toward the support, (2) adjust the movement of the newly developed leaf as a function of the previous one, and (3) reduce possible errors in the establishment of contact points for clasping the support (Guerra et al., 2024). This strategy characterized by a continuous cycle of information exchange between the environment and the plant might allow plants to use the information from the previous leaf such as input signals and tracking errors, to develop a successful and controlled clasping movement toward the support. Therefore, even in plants, the speed characterizing the execution of an action and the time window in which it is executed represent key factors that enable the success of the plant.

**Fig. 4:**
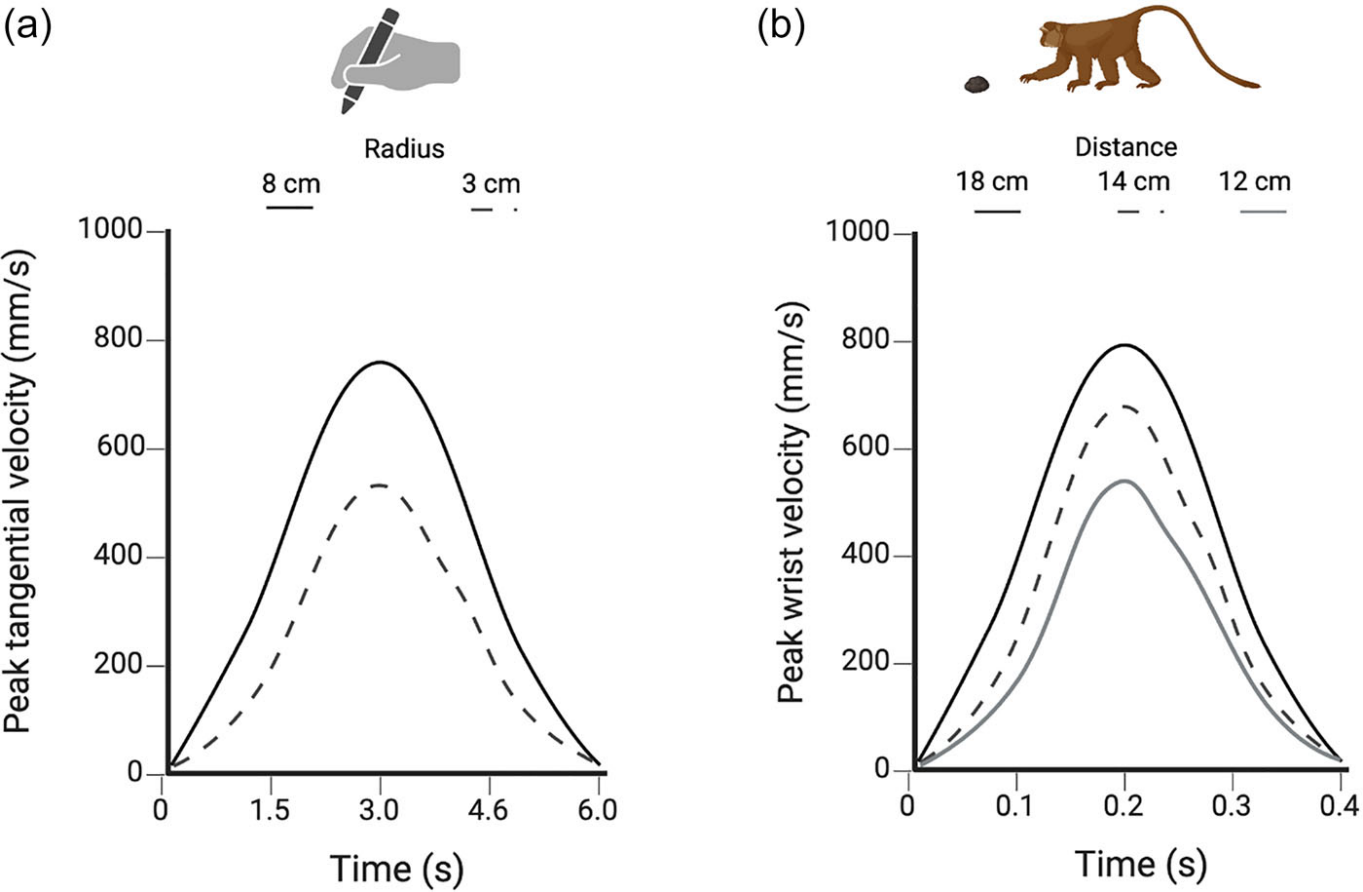
Comparative studies on isochrony. Graphical representation of the variations in peak velocity (mm/s) from Viviani and McCollum (1983; A) and Sartori, Camperio‐Ciani, Bulgheroni, and Castiello (2013; B). Panel A shows the peak velocity of humans during the drawing of circles with different radius diameters, while panel B shows the peak velocity of the macaques’ wrist during the reach‐to‐grasp movement toward objects located at different distances. The graphs show the spontaneous tendency to increase the speed of the movements depending on the task and the properties of the to‐be‐grasped object in order to keep the execution time approximately constant. Abbreviations: cm, centimeters; mm, millimeters; s, seconds.

In the context of these studies, it is important to consider the relevance of the temporal dimension. For instance, plants tend to maintain a constant duration of circumnutation by scaling velocity (Wang et al., 2023). Evidence has shown that plants tend to apply the principle of isochrony as many other living organisms (cf. Viviani & McCollum, 1983; Fig. 4).

Given the importance of isochrony for plants, some clarifications are in order. At a general level, “isochrony” means “to have the same time.” Isochrony has been traced back to different forms of regularity identified in processes and movements, investigated at various levels and contexts. The principle of isochrony refers to a phenomenon of motor control in which the velocity of a movement is proportional to its trajectory so that the execution time remains constant. For example, studies of human writing movements have shown that it takes the same time to write a letter or word of different sizes, implying that there are proportional changes in the speed of movement execution (Lacquaniti, Terzuolo, & Viviani, 1983). Furthermore, in linguistics, isochronism is the characteristic of languages to rhythmically mark time in equal parts or of similar duration. The human ear is able to perceive words that have the same number of syllables, although with a different number of phonemes (cf. Ravignani, Honing, & Kotz, 2017). From a physiological point of view, many bodily processes have significant regularities or isochronous elements. Examples are heartbeat, breathing, and locomotion (cf. Ravignani & Madison, 2017). Historically, from a physical‐mathematical perspective, the Galilean observations of the early 1600s on the properties of the *pendulum* have played an important role in the development of a concept of temporal regularity and measurements. Galileo, instead of seeing the *pendulum* simply as an oscillating body, framed it as a process capable of measuring time (Ariotti, 1968). Subsequently, examples of isochronous processes have been offered in physics, as in the case of atomic clocks, based on oscillations relative to atomic activity occurring at known frequencies (cf. Strogatz, 2003). From an information theory perspective (cf. MacKay, 2003), an isochronous signal minimizes the entropy of a signal, since it is composed of an easily predictable and determined sequence, that is, with a known rate of signal repetition.

To us, the most interesting aspect concerns the bioecological roots of the propensity to isochrony (cf. Buzsaki, 2006). In our species, for instance, this is visible from an early age, given the ability of infants and young children to react differently to isochronous sequences or not (cf. Honing, Ladinig, Háden, & Winkler, 2009; Jeannerod, 1984). The relationship between linear extension (length of a movement) and compensation in terms of speed is a characteristic that concerns different types of human behavior and actions, such as hand and arm movements, kicking activity in infants, weightlifting, typing, writing (cf. Sartori et al., 2013). Compared to the case of writing, for example, it has been observed that forgers, in an attempt to perform false signatures, produce ineffective movements that violate isochrony, with duration‐amplitude coefficients significantly higher than authentic signatures (Caligiuri, Mohammed, Found, & Rogers, 2012).

Isochronous behaviors, on the other hand, are not an exclusive feature of our species, they are also found in many other living species (cf. Schaeffer et al., 2024). Some species that emit isochronous signals externally (the so‐called “isochronous species”) are, for example, crickets, frogs, fireflies, birds, crabs, and marine mammals (cf. Ravignani & Madison, 2017). Furthermore, it has been observed that macaques show a spontaneous tendency to increase the speed of their reach‐to‐grasp movements depending on the distance of the object to be grasped, to keep the execution time approximately constant (Sartori et al., 2013; Fig. 4B).

Therefore, from a biological point of view, the isochrony of movement can be linked to the principles of minimization that describe how organisms try to obtain maximum effectiveness with minimum effort during the execution of a movement directed to a goal (Caligiuri et al., 2012). In this case, the principle of isochrony represents a compensation mechanism, which states that the speed of the movement increases according to the linear extension of the trajectory to keep the execution time approximately constant. The time required to perform an action remains constant, while the speed is proportional to the distance to be covered (Sartori et al., 2013).

**Fig. 5:**
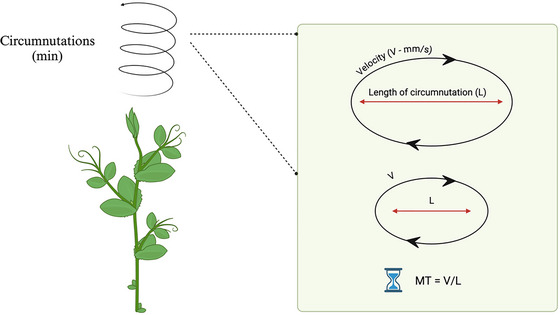
The principle of isochrony in plants. Graphical representation of the principle of isochrony in plants. Plants perform circular movements (i.e., circumnutation) during their growth. Plants tend to maintain a constant movement time duration (MT) and scale velocity (V) in order to cover longer distances (longer circumnutation length—L). This strategy allows plants to program the patterning of circumnutation during the ascend‐to‐clasp movement in different environmental contexts. Abbreviations: L, length of the circumnutation; mm, millimeters; MT, movement time duration; s, seconds; V, velocity.

Back to plants, it has been observed that even in plant movements, there is evidence of isochrony. It has been demonstrated that plants tend to maintain the duration of the movement constant to cover longer circumnutations trajectories by scaling the speed of their movements during the approaching and clasping of a potential support (Wang et al., 2023; Bonato et al., 2024; Fig. 5).

**Fig. 6:**
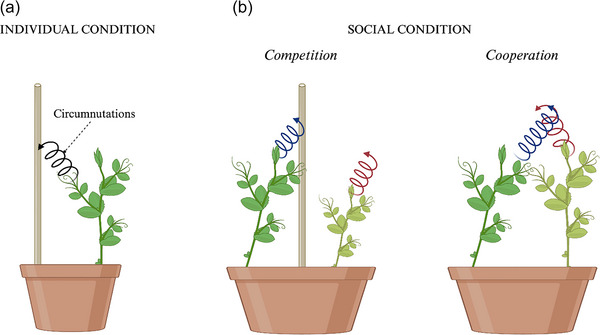
Contextual variation in plant movement behavior. Graphical illustration of the experimental conditions from Bonato et al. (2023; 2024). (A) Individual condition. Plants were tested in isolation during the ascending and clasping of a potential support. (B) Social condition. In the social condition, plants were placed in the same pot with a support in the middle (competition) or without a support (cooperation). In the presence of a support, plants showed two distinguished behavior: the “winner” plant exhibited a circumnutation pattern (blue spiral line) toward the support, while the “loser” plant showed a pattern of circumnutation (red spiral line) along the vertical axis without manifesting any orientation toward the support. In the absence of a potential support, plants coordinated their circumnutation pattern to support each other and achieve a common goal (i.e., reach the greatest exposure to the light).

At stake is the importance of the link between the type of movement, its speed, and the context in which the movement is performed. Other aspects, that depict the importance of time for plants, emerge when looking at the “social” side of plants’ behavior. In this regard, Bonato et al. (2023) studied the kinematics of the movement of pea plants placed in the same pot competing for access to a potential support (Fig. 6A,B). It emerged that acting in the presence of another plant influences how the movement is performed.

Two specific behavioral attitudes were distinguished: a “submissive” behavior characterized by a slower movement pertaining to the so‐called “loser” plant, the plant losing the competition for the support, and a “forceful” behavior characterized by a faster movement pertaining to the so‐called “winner” plant, the plant winning the competition for the support. This suggested that even in the plant case it is essential to consider the strategies of other organisms (cf. McNickle & Dybzinski, 2013). Therefore, together with other factors, modulation of response time and motor patterns give information about behavioral differences in plants, studied under both individual and social conditions. Turning to cooperation, it was investigated how two pea plants, in the absence of a support, wrap themselves and coordinate their movements to support each other to gain access to light (Bonato et al., 2024; Fig. 6B). The plants coordinate their movement in terms of correlating in time the kinematic parameterization of their movement to facilitate intertwining (Fig. 6B).

## 4 Conclusions: Some considerations and open questions

Here, we provide an overview of how important is to study time in living systems, with particular reference to how time is managed by plants. We feel this is relevant both for a better understanding of these organisms and for broadening our horizons on the different forms of cognition present in nature (Bianchi, 2024a; 2024b).

From a theoretical point of view, it is extremely interesting to be able to understand how different living systems, which use different behavioral strategies with different action times, can be unified through the concept of time to solve common survival problems. This means that the time dimension, although declined in different ways, can be the common denominator for adapting and solving vital challenges.

In this respect, a variety of open questions call for deeper insights and require further exploration.

For instance, reflecting on the “slowness of life” in plants—their different rates of growth, modes of communication, and movements in response to environmental challenges—encourages us to consider, on one hand, the universal need of all living beings to solve survival problems, and on the other hand, the relativity of behaviors shaped by distinct bodily structures and ecological needs (specific to each species and individual organism). What we call the “slowness” of plant behavior is such when compared to our lifetimes (it is good to remember that some processes, such as intra‐ and intercellular ones, are fast). The relative slowness of signaling in plants results from physical and biochemical constraints on molecular movement and diffusion within tissues, and from the absence of a nervous system, which in animals enables rapid signaling, integration of electrochemical signals, and coordination of movement. The slowness of plant life could be linked to the fact that these organisms are rooted in their context of growth and their forms of communication concern mainly (but not exclusively) the ability to recognize and synthesize chemical compounds, something that makes them able to “know” with a certain degree of accuracy what they have around them, without the need to move and consequently hurry up to escape threats and dangers (cf. Bianchi, 2021). From a biotemporal perspective, slowness is a relational concept that cannot be considered in absolute or independent terms, as it always implies an element of comparison.

These considerations lead us to reflect on the difference between the “actual” time of plant life and the time we can concretely observe, based on our perceptual‐cognitive constraints and through the use of techniques such as time‐lapse that make plant movements visible and understandable to us. One may ask: what does a minute or an hour of “our time” mean for a particular plant species? Is the time derived from time‐lapse analyses a realistic indication of the life, communication, and behavior of plants? Has it the same value of when we slow down the movement of animals for picking up subtle features? This is an aspect that should be verified and pursued in further research.

Moreover, it will be interesting to understand how the different plant communication modes follow different times and to what extent they can be integrated with each other (cf. Bianchi et al., 2025). For instance: do the forms of plant signaling related to chemical emissions, movement, or shape modification follow different times? At what level and in what ways can they be received and “understood” by other organisms? Do they have an intraspecific or even interspecific scope? Is there a sharing of the same “ecological time” in the interspecific communication of organisms belonging to different species and kingdoms? (cf. Bianchi, 2022).

Other interesting questions concern a better investigation of whether and exactly how plants present different communication times at different stages of individual development, and how this aspect varies in longer‐lived species (cf. Thomas, 2002).

In a more specific sense, research on the aspects of isochrony could help to understand how the perception of the duration, and regularity of a process can be broken, thus changing the meaning of that specific behavior or signal. It is a question of understanding if, and to what extent, even in plants, there is an ability to use and modulate sequences of signals and movements, and whether this helps to understand what a signal represents, and whether this can be communicated to other species. It is, therefore, a question of understanding whether the disruption of predictability or modification of the regularity of a movement or behavioral pattern can be interpreted as a form of intentional communication (cf. Merker, Madison, & Eckerdal, 2009).

A question that arises in this regard is whether the principle of isochrony in the context of bioecological and behavioral research can be understood as a common principle to explain the presence of shared regularities and patterns across *taxa*.

Other interesting aspects concern whether there is an equivalent of “turn‐taking” in the forms of plant communication (e.g., in the emission and perception mechanisms of organic molecules). That is, whether there are species‐specific times in the signaling and in what circumstances the times vary. In our species, turn‐taking is the phenomenon of temporal scanning according to which, even if not perfectly isochronous, and variable between individuals, the interlocutors are able to interact effectively in verbal communication, avoiding their expressions overlap (cf. Levinson & Torreira, 2015). As we know from communication studies, the meaning of a transmitted message could lie in the emission of the signal itself (the concept underlying “signal signaling”) (cf. Scott‐Phillips, Kirby, & Ritchie, 2009). The message of an isochronous model, as opposed to what happens in referential communication, which refers to a state of affairs in reality, is precisely its “isochronicity” (cf. Ravignani & Madison, 2017).

Another aspect that could be deepened is whether for vitally important events that “capture” the attention the most (cf. Ivry & Schlerf, 2008), involving a greater expenditure of energy (cf. Jamadar et al., 2025), plants can change their electrical and chemical signaling patterns and whether their rates of growth, response, and information processing vary (cf. Parise et al., 2023).

In the name of a comparative and processual approach, one of the upcoming challenges is to deepen more—thanks to the observation of the forms of intra and interspecies movement and communication—the ability of plants to perceive the passage of time, the duration, and regularity of phenomena, the ability to “read” the behavior of other organisms to solve survival problems. In this way, it will be possible to understand how much of this is exclusive to the plant kingdom, how much is species‐specific, how much is related to the individual development of a single organism, and how much, finally, is shared with other species.

For now, it seems that without a sense of time, being cognitively active in the world may not be possible. Where there is transformation and movement, there must be the possibility of keeping track of the past and predicting, at least from a functional‐agentive perspective, what will happen in the future, more recent or more remote.

## Author contributions

Conceptualization: M.B. and U.C.; funding acquisition and project administration: U.C.; writing—original draft: M.B.; writing—review and editing: M.B., S.G., B.B., S.A., L.R., V.S., M.D, and U.C. All authors have read and agreed to the final version of the manuscript.

## Funding

The study was funded by the European Union (ERC, ROOMors, Grant Number 101096728) to U.C. Views and opinions expressed are those of the author(s) only and do not necessarily reflect those of the European Union or the European Research Council Executive Agency. Neither the European Union nor the granting authority can be held responsible for them.

## Data Availability

No data were used for the research described in the article.
